# Adenosine 2B Receptor Activation Reduces Myocardial Reperfusion Injury by Promoting Anti-Inflammatory Macrophages Differentiation via PI3K/Akt Pathway

**DOI:** 10.1155/2015/585297

**Published:** 2015-06-16

**Authors:** Yikui Tian, Bryan A. Piras, Irving L. Kron, Brent A. French, Zequan Yang

**Affiliations:** ^1^Department of Surgery, University of Virginia, P.O. Box 800709, Charlottesville, VA 22908, USA; ^2^Department of Cardiovascular Surgery, Tianjin Medical University General Hospital, Tianjin 300052, China; ^3^Department of Biomedical Engineering, University of Virginia, Charlottesville, VA 22908, USA

## Abstract

*Background*. Activation of the adenosine A_2B_ receptor (A_2B_R) can reduce myocardial ischemia/reperfusion (IR) injury. However, the mechanism underlying the A_2B_R-mediated cardioprotection is less clear. The present study was designed to investigate the potential mechanisms of cardioprotection mediated by A_2B_R.* Methods and Results*. C57BL/6 mice underwent 40-minute ischemia and 60-minute reperfusion. ATL-801, a potent selective A_2B_R antagonist, could not block ischemic preconditioning induced protection. BAY 60-6583, a highly selective A_2B_R agonist, significantly reduced myocardial infarct size, and its protective effect could be blocked by either ATL-801 or wortmannin. BAY 60-6583 increased phosphorylated Akt (p-Akt) levels in the heart at 10 min of reperfusion, and this phosphorylation could also be blocked by ATL-801 or wortmannin. Furthermore, BAY 60-6583 significantly increased M2 macrophages and decreased M1 macrophage and neutrophils infiltration in reperfused hearts, which also could be blocked by wortmannin. Meanwhile, confocal imaging studies showed that the majority of Akt phosphorylation in the heart was colocalized to CD206+ cells in both control and BAY 60-6583 pretreated hearts.* Conclusion*. Our results indicated that pretreatment with BAY 60-6583 protects the heart against myocardial IR injury by its anti-inflammatory effects, probably by modulating macrophages phenotype switching via a PI3K/Akt pathway.

## 1. Introduction

The adenosine receptor (AR) family comprises four subtypes: A_1_, A_2A_, A_2B_, and A_3_. They are widely distributed in mammalian species. The A_2B_R is the fourth AR subtype identified, and to date there is much less information available on the precise role of this receptor compared to the other AR subtypes. This notwithstanding an increasing body of evidence demonstrates that activation of A_2B_Rs by the selective A_2B_R agonist, BAY 60-6583, either before ischemia [1, 2] or before reperfusion [3, 4] reduces myocardial infarct size. Thus, pharmaceutical preconditioning with A_2B_R activation has been shown to protect against myocardial I/R injury, but the role of A_2B_Rs in IPC remains controversial.

Using an* in vivo* mouse model of IPC and genetic knockouts of ARs, Eckle et al. challenged the mechanism of A_1_R-mediated IPC [5–7] by proposing that A_2B_Rs, not A_1_Rs, are essential in mediating IPC via an adenosine signaling pathway involving ecto-5′-nucleotidase [1]. However, Maas et al. reported that A_2B_R activation is not required for IPC in either rat or mouse models. Nevertheless, they did find that pretreatment prior to index ischemia with an A_2B_R agonist did reduce IR injury, but to a lesser degree compared to that of IPC [2]. Interestingly, another study reported by Eckle's group [8] recently demonstrated that BAY 60-6583 protected the heart against IR injury not by acting on cardiomyocytes but by activating the A_2B_R on bone marrow derived cells and reducing inflammatory cell infiltration in reperfused heart. The discrepancies between these published results call for more studies to clarify the role of A_2B_Rs in IPC and to explore the mechanisms underlying the A_2B_R-mediated cardioprotection against IR injury.

By using a well-established intact mouse model with myocardial IR injury, the current study was undertaken to further define the roles of the A_2B_R in IPC and its anti-inflammatory effects during myocardial IR injury.

## 2. Materials and Methods

This study conformed to the Guide for the Care and Use of Laboratory Animals published by the National Institutes of Health (Eighth Edition, revised 2011) and was conducted under protocols approved by the University of Virginia's Institutional Animal Care and Use Committee.

### 2.1. Agents and Chemicals

2,3,5-Triphenyltetrazolium chloride (TTC) and wortmannin were purchased from Sigma-Aldrich (St. Louis, MO). Phthalo blue was purchased from Heucotech Ltd. (Fairless Hills, PA). BAY 60-6583 was purchased from Tocris Bioscience (Bristol, UK). ATL-801 was kindly provided by Lewis and Clark Pharmaceuticals, Inc. (Charlottesville, VA). Antibodies against phospho-Akt and total Akt were purchased from Cell Signaling Technology (Beverly, MA). Ly-6B.2 and CD206 antibodies for neutrophils staining were from ABDSerotec (Oxford, UK). CD45 antibody was from BD Biosciences (San Jose, CA). All fluorochrome-conjugated secondary antibodies and Prolong Gold antifade reagent with DAPI were from Life Technologies (Grand Island, NY).

### 2.2. Animals and Experimental Protocol

**Figure 1:**
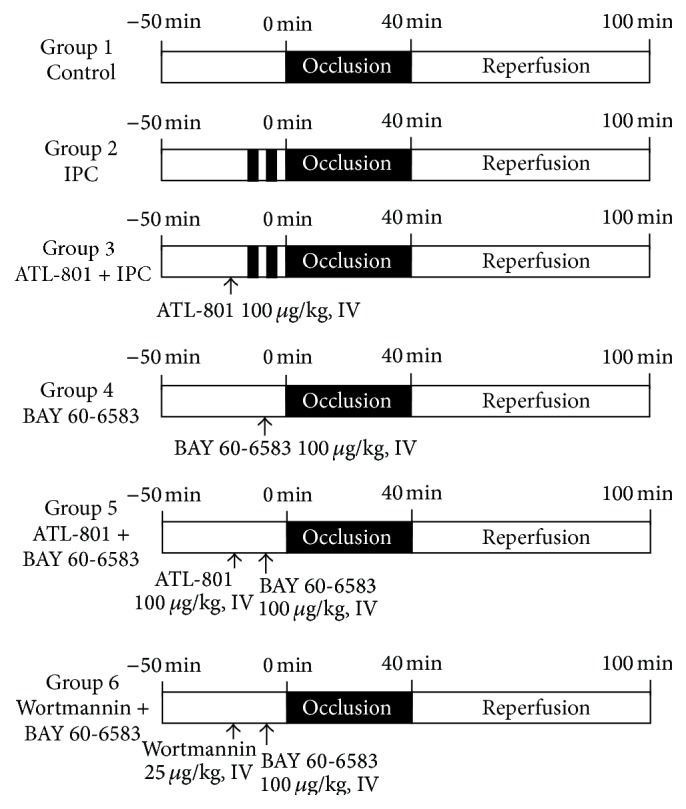
Experimental protocol. All mice underwent 40 min ischemia followed by 60 min reperfusion. At the end of experiments, hearts were harvested for TTC staining to determine the myocardial infarct size. IPC: ischemic preconditioning.

C57BL/6 mice (9-13 weeks old, purchased from Jackson Laboratories) were assigned to 6 different groups as shown in Figure 1. These mice underwent 40 min of LAD occlusion followed by 60 min of reperfusion with or without IPC. IPC was applied to mice with two cycles of 5- minute ischemia and 5-minute reperfusion. In the treated groups, BAY 60-6583 (100 *μ*g/kg, iv) was administered 15 minutes before index LAD occlusion. ATL-801 (100 *μ*g/kg, iv) was administered 5 minutes before either BAY 60-6583 injection or IPC. Wortmannin (25 *μ*g/kg, iv) was administered 5 minutes before BAY 60-6583 injection. The doses of the BAY 60-6583, ATL-801, and wortmannin used in this study were equal to or less than those used in the literature, which reported no significant effect on hemodynamics. Additional 3 mice from each group were undergoing sham surgery. We monitored heart rate and found no significant difference when compared to control groups (Table 1). The hearts were harvested at the end of the experiments for infarct size measurement or immunostaining. Effects of BAY 60-6583 on phosphorylated-Akt (p-Akt) levels in hearts which underwent 40 minutes of ischemia followed by 10 minutes of reperfusion were tested by western blot. The hearts from BAY 60-6583 + ATL-801 or BAY 60-6583 + wortmannin groups were also harvested for p-Akt analysis.

### 2.3. Myocardial Ischemia/Reperfusion Injury and Measurement of Infarct Size

Mice were subjected to 40 minutes of coronary occlusion followed by 60 minutes of reperfusion as detailed previously [5, 9–11]. Briefly, mice were anesthetized with sodium pentobarbital (100 mg/kg i.p.) and orally intubated. Artificial respiration was maintained with a FiO_2_ of 0.80, 100 strokes per minute, and a 0.2 to 0.5 mL stroke volume. The heart was exposed through a left thoracotomy. A 7-0 silk suture was placed around LAD at a level 1 mm inferior to the left auricle and a miniature balloon occluder fashioned from Microbore Tygon tubing (Small Parts Inc., Seattle, WA) was affixed over the LAD. Ischemia and reperfusion were induced by inflating or deflating the balloon, respectively. ECG was monitored perioperatively using PowerLab instrumentation (ADInstruments, Colorado Springs, CO). The mice were euthanized 60 minutes after reperfusion, and the hearts were cannulated through the ascending aorta for perfusion with 3 to 4 mL of 1.0% TTC. The LAD was then reoccluded with the same suture used for coronary occlusion prior to 10% Phthalo blue perfusion to determine risk region (RR). The left ventricle was then cut into 5 to 7 transverse slices that were weighed and digitally photographed to determine infarct size as a percent of RR.

### 2.4. Western Blot Analysis

The total protein was extracted from the indicated experimental groups using RIPA buffer and protein concentration was determined by BCA protein assay (Thermo Scientific, Rockford, IL). All western blots were performed according to standard procedures. Twenty micrograms of protein was separated by 10% SDS-PAGE. After transfer, nitrocellulose membranes (BioRad, Hercules, CA) were probed with primary antibodies against total Akt (t-Akt) or S473 (p-Akt) at a 1 : 2,000 dilution and secondary antibodies (Promega, Madison, WI) at a 1 : 5,000 dilution in blocking solution (0.5% BSA in TBS-T). Proteins were visualized with enhanced chemiluminescent substrate (Thermo Scientific, Rockford, IL), followed by densitometry analysis using Fluorchem 8900 imaging system (Alpha Innotech, Santa Clara, CA).

### 2.5. Immunohistochemistry for Neutrophils

The hearts were harvested and immediately fixed in 4% paraformaldehyde in PBS (pH 7.4) for paraffin embedding. Paraffin-embedded sections (5 *μ*m) were rehydrated and incubated with 1% hydrogen peroxide. After being rinsed in PBS, the sections were incubated with 10% blocking serum. Immunostaining was performed with rat anti-mouse Ly-6B.2 antibody. Biotinylated secondary antibody was then applied for 1 hour at room temperature. After incubation with avidin-biotin complex, immunoreactivity was visualized by incubating the sections with 3,3-diaminobenzidine tetrahydrochloride to produce a brown precipitate.

### 2.6. Immunofluorescence Staining

In order to determine the localization of p-Akt expression, immunofluorescence staining was performed on hearts after 40 minutes of ischemia and 10 minutes of reperfusion in control or BAY 60-6583 treated groups. Cardiac macrophage polarization was detected after 60 minutes of reperfusion by defining M1 and M2 subsets of macrophages. Hearts were fixed in 4% paraformaldehyde in PBS (pH 7.4) for 1 hour at room temperature and then incubated in 30% sucrose overnight at 4°C before freezing in OCT. Frozen sections were cut and permeabilized by 0.3% TritonX-100 in PBS. After blocking with 10% normal serum for 1 hour, specimens were colabeled with anti-p-Akt and anti-CD45 or anti-CD206 antibody overnight at 4°C for colocalization detection. For macrophage staining, specimens were incubated with CD86 or CD163 antibodies to identify the M1 and M2 macrophages, respectively. After washing, sections were incubated with a mixture of Alexa Fluor 488- and Alexa Fluor 594-conjugated secondary antibodies for 1 hour. Prolong Gold antifade reagent with DAPI was used to mount the specimens. All images were acquired under the same parameters for each fluorochrome using an Olympus BX-41 Microscope (Olympus, America, Inc., Center Valley, PA) with a Retiga-2000R camera (QImaging, Surrey, BC). Further image processing was performed using Image J software (NIH).

### 2.7. Statistical Analysis

All data are presented as the mean ± SEM (standard error of the mean). Peri-ischemic heart rate changes were analyzed using a repeated measures ANOVA followed by Bonferroni pairwise comparisons. All other data were compared using one-way ANOVA followed by *t*-test for unpaired data with Bonferroni correction.

## 3. Results

### 3.1. Perioperative Heart Rate Changes

Table 1 shows changes in heart rate before, during, and after LAD occlusion. Consistent with previous reports, heart rate was increased significantly after LAD occlusion and remained elevated until early reperfusion compared with baseline in all groups. There was no significant difference in heart rates between control and treated mice.

### 3.2. Role of A_2B_R in IPC-Induced Infarct-Sparing Effect

**Figure 2:**
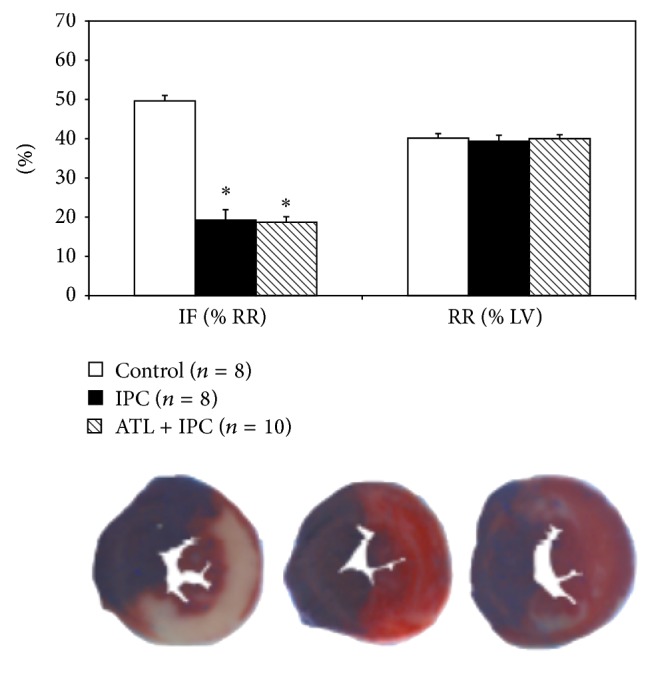
Role of A_2B_R antagonist in ischemic preconditioning. Pretreating mice with ATL-801, an A_2B_R selective antagonist, did not block the cardioprotective effect of IPC as compared with the control group (^*^ *P* < 0.05). IF: infarct size; RR: risk region; LV: left ventricle; IPC: ischemic preconditioning; ATL: ATL-801.

Three groups of mice which underwent 40 minutes of LAD occlusion followed by 60 minutes of reperfusion were designed to investigate the role of A_2B_R in IPC phenomenon. There was no significant difference of risk region (RR) among the three groups. Infarct size in the IPC-treated group (19.2 ± 2.7% of RR) was significantly reduced compared with the control group (49.6 ± 1.4% of RR, *P* < 0.05). Administration of A_2B_R selective antagonist, ATL-801, 5 minutes before IPC could not block the cardioprotective effects (18.7 ± 1.4% versus 19.2 ± 2.7% of RR, *P* > 0.05) (Figure 2).

### 3.3. Activation of A_2B_R Reduced Myocardial IR Injury Is Mediated by PI3K/Akt Pathway

**Figure 3:**
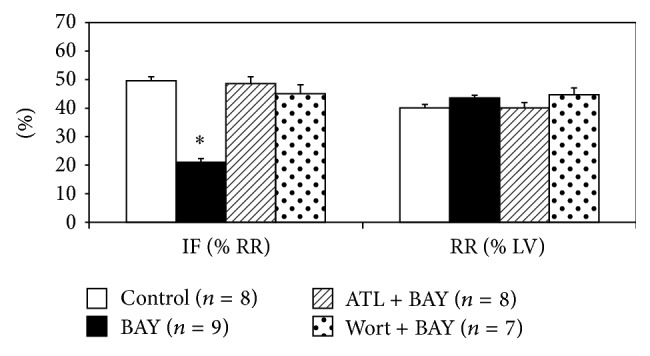
Role of A_2B_R in myocardial IR injury. Pretreating mice with BAY 60-6583, a potent A_2B_R agonist, reduced myocardial infarct size, an effect that was abolished by either ATL-801 or the PI3K inhibitor: wortmannin. IF: infarct size; RR: risk region; BAY: BAY 60-6583; ATL: ATL-801; Wort: wortmannin. ^*^ *P* < 0.05 compared with control group.

**Figure 4:**
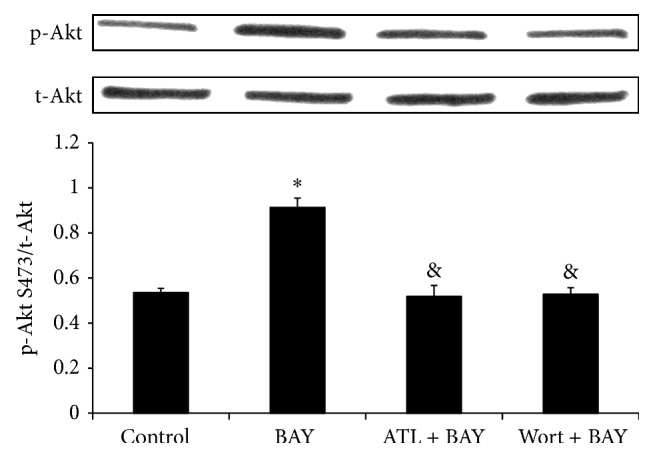
Phospho-Akt to total Akt ratio in heart tissue. The ratio of phospho-Akt S473 to total Akt (the bar graph) was measured by densitometry, where the total Akt inputs were normalized to 1. BAY 60-6583 pretreatment increased p-Akt level after 10 minutes of reperfusion, which was blocked by either ATL-801 or wortmannin. BAY: BAY 60-6583; ATL: ATL-801; Wort: wortmannin. ^*^ *P* < 0.05 compared with control group; ^&^ *P* < 0.05 compared with BAY group.

BAY 60-6583 had no effects on heart rate during the peri-ischemic phase (Table 1). Administration of BAY 60-6583 15 minutes before LAD occlusion had significant infarct-sparing effects against myocardial reperfusion injury (21.0 ± 1.3% versus 49.6 ± 1.4% of RR, *P* < 0.05). Pretreating the mice with ATL-801 before BAY 60-6583 administration completely blocked the cardioprotective effect (48.6 ± 2.4% of RR, *P* < 0.05, compared with BAY group) (Figure 3). As shown in Figure 3, the infarct-sparing effect of BAY 60-6583 was completely abrogated by wortmannin, a selective PI3K inhibitor, administered 5 minutes before BAY administration (45.0 ± 3.2% of RR, *P* < 0.05 compared with BAY group). Furthermore, western blot results showed that BAY 60-6583 significantly increased p-Akt levels in heart tissue undergoing 40 minutes of ischemia and 10 minutes of reperfusion. This effect was completely abolished by pretreating the mice with either ATL-801 or wortmannin 5 minutes before BAY 60-6583 administration (Figure 4).

### 3.4. Administration of A_2B_R Agonist Presents Local Anti-Inflammatory Effects after Myocardial IR Injury

**Figure 5:**
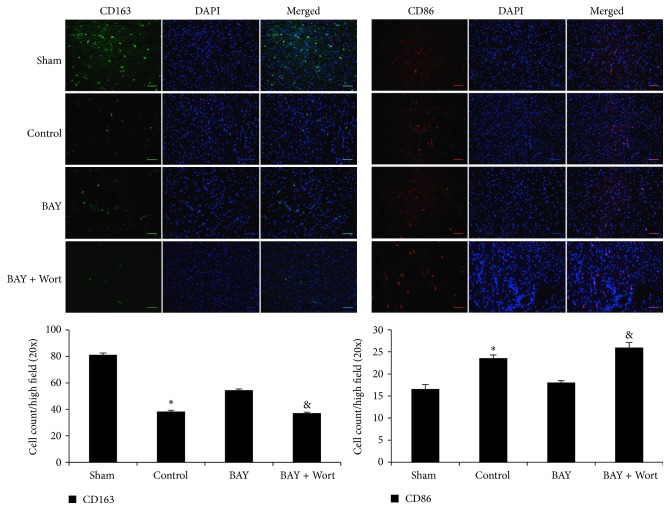
Cardiac macrophages polarization after IR injury. There are mainly M2 subsets macrophages in normal heart. IR reduced the number of M2 subset (CD163, green staining) macrophages and increased M1 subset (CD86, red staining). BAY 60-6583 pretreatment significantly restored the macrophage polarization during reperfusion. Wortmannin, a PI3K inhibitor, blocked the effects of BAY 60-6583. DAPI was used to stain the nuclei. Scale bar: 50 *μ*m. IR: ischemia and reperfusion injury; BAY: BAY 60-6583; Wort: wortmannin. ^*^ *P* < 0.05 compared with sham or BAY groups; ^&^ *P* < 0.05 compared with BAY groups.

**Figure 6:**
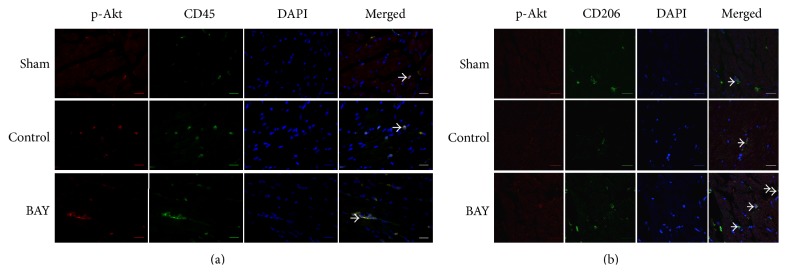
Confocal staining of p-Akt and immune cells in the heart. Hearts were harvested from mice with 40 min/10 min IR injury for staining to determine the localization of p-Akt expression. (a) In the ischemic area or lower anterior wall of the left ventricle, higher p-Akt expression (red staining) was colocalized with CD45 signal (green staining) in all groups. Scale bar: 20 *μ*m. (b) The majority of strong p-Akt expression (red staining) was colocated with CD206 expression (green staining), which is a specific marker of M2 macrophages. Scale bar: 20 *μ*m. Arrows indicate coexpression of p-Akt and CD45 or CD206. 4′,6-Diamidino-2-phenylindole (DAPI, blue staining) was used to stain the nuclei. IR: ischemia and reperfusion injury; BAY: BAY 60-6583.

**Figure 7:**
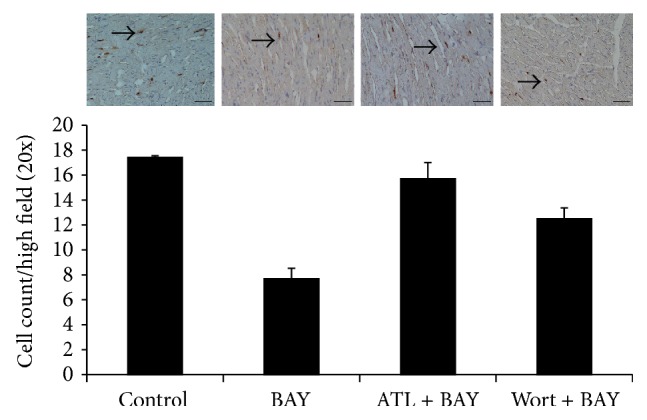
Neutrophils infiltration after IR injury. BAY 60-6583 significantly reduced neutrophil infiltration (brown staining) after IR injury, which could be blocked by either ATL-801 or wortmannin. Scale bar: 50 *μ*m. Arrows indicate neutrophils. BAY: BAY 60-6583; ATL: ATL-801; Wort: wortmannin. ^*^ *P* < 0.05 compared with other groups.

It has been reported that activation of A_2B_R has anti-inflammatory effects by promoting macrophages phenotype switching. Also activated PI3K/Akt pathway has been shown to modulate macrophages to M2 anti-inflammatory subset. Thus, we hypothesized that BAY 60-6583 may protect the heart by regulating cardiac macrophages phenotype via PI3K/Akt pathway and presenting anti-inflammatory effects. Immunofluorescence staining was used to identify macrophage subsets. In sham mouse heart, there were few M1 macrophages (CD86+) but more M2 macrophages (CD163+). IR significantly increased the M1 and decreased the M2 macrophages number. Compared with the IR group, BAY 60-6583 restored the macrophage polarization to a M2 phenotype (Figure 5), which could also be abolished by wortmannin. In addition, confocal staining results showed that remarkable p-Akt expressions were colocalized with CD45+ cells (Figure 6(a)). In order to further identify these CD45+ cells, we performed additional confocal microscopy studies on tissue sections immunostained with p-Akt and CD206 antibodies, another specific marker of M2 macrophages. As shown in Figure 6(b), although a few of the p-Akt+ cells were CD206 negative, the majority of the p-Akt expression colocalized with M2 macrophages. These results suggest that BAY 60-6583 may protect the heart by promoting macrophage phenotype switching to anti-inflammatory subsets via the PI3K/Akt pathway. Immunohistochemistry was used to stain neutrophils in hearts undergoing 40 minutes of ischemia and 60 minutes of reperfusion. When pretreated with BAY 60-6583 before IR, neutrophil infiltration was significantly reduced. Consistent with the infarct size results, this anti-inflammatory effect of BAY 60-6583 could be blocked by both ATL-801 and wortmannin, respectively (Figure 7).

## 4. Discussion

For over a decade, abundant evidence has accumulated to demonstrate that brief periods of myocardial ischemia (IPC) activate the A1R to protect the heart against the subsequent prolonged ischemia [5, 12–15]. Activation of A1Rs triggers the survival signal transduction pathway through the enhanced phosphorylation of Akt [15–17]. This event mostly likely occurs inside cardiomyocytes, thus rendering them more resistant to the subsequent ischemic insult [5, 12]. Eckle and colleagues [1] recently proposed that cardioprotective effect of IPC was mediated via CD73-dependent generation of extracellular adenosine and signaling through the A_2B_R, the lowest-affinity receptor among the 4 subtypes of adenosine receptor. A_2B_RKO mice demonstrate increased susceptibility to acute IR injury and are not protected by IPC. However, Maas and colleagues evaluated the role of A_2B_R activation or inhibition in both* ex vivo* and* in vivo* mouse/rat models with myocardial IR injury and found that IPC-induced cardioprotection was not dependent upon A_2B_R activation and also existed in a different strain of A_2B_RKO mice [2]. It should be noted that the lowest affinity for adenosine could make A_2B_R be the last receptor activated by endogenous adenosine, which is the core mechanism of IPC [18], although A_2B_R might be sensitized by PKC at reperfusion phase [19]. Also A_2B_R expression at the mRNA level is very low in the heart when compared to other organs [20] or other adenosine receptors [21]. Moreover, there has been no physical evidence so far to demonstrate that A_2B_Rs can be detected on the sarcolemma of cardiomyocytes, but increased A_2B_R mRNA expression has been reported after IR injury [22], which may be derived from infiltrating inflammatory cells. By employing the same* in vivo* mouse model we used previously [9, 11, 17, 23], the current study demonstrated activation of A_2B_Rs before ischemia reduced myocardial infarct size, probably by inhibiting inflammatory responses during reperfusion, not by preconditioning cardiomyocytes (Figures 2 and 3).

Accumulating evidences have shown that inflammatory responses play important roles during myocardial reperfusion injury [10, 11, 24, 25]. A_2B_R has been shown to have anti-inflammatory effects in several* in vitro* and* in vivo* animal models. Yang and colleagues [26] found that A_2B_RKO mice present proinflammatory phenotype. They found that more inflammatory cytokines, such as TNF-alpha and IL-6, increased in serum of A_2B_RKO mice compared with wild-type mice. Konrad and colleagues [27] reported that BAY 60-6583, a specific A_2B_R agonist, acts on hematopoietic cells to inhibit PMN migration into lung interstitium. BAY 60-6583 also has been shown to inhibit TNF-alpha secretion from macrophages after vascular injury [28]. Consistent with these studies, we found that BAY 60-6583 significantly promoted macrophages phenotype switching to a M2 (anti-inflammatory) subset and reduced neutrophils infiltration after myocardial IR injury (Figures 5 and 7). Indeed, A_2B_R has been shown to be able to modulate macrophage phenotype to an anti-inflammatory M2 subset and increase IL-10 expression in both cultured macrophage and dendritic cell [29, 30], which are consistent with our findings that A_2B_R agonist promoted the cardiac macrophage transforming to an anti-inflammatory M2 phenotype. Indeed, previous studies have shown that BAY 60-6583 provided potent cardioprotection in a Krebs-perfused isolated heart model. However, it is worthwhile to note that there actually are several residential macrophages and dendritic cells in the heart, which may mediate the cardioprotection of the A_2B_R agonist. Further study is warranted to investigate the mechanism of how these residential immune cells regulate myocardial IR injury.

Activation of PI3K/Akt pathway in immune cells has been reported providing anti-inflammatory effect by upregulating IL-10 expression [31, 32]. The relationship between A_2B_R activation and PI3K/Akt pathway is not well established. Kuno and colleagues [3] reported that activation of A_2B_R increased p-Akt levels in a rabbit model, although a nonselective adenosine receptor agonist was used. It is also reported that activation of A_2B_R contributes to PI3K/Akt activation and subsequent eNOS phosphorylation in penile endothelia [33]. In present study, we found that after 40 minutes of ischemia and 10 minutes of reperfusion, phosphorylation of Akt in the heart was significantly increased in mice treated with A_2B_R agonist, which could be blocked by either ATL-801, a selective A_2B_R antagonist, or wortmannin, a PI3K inhibitor (Figure 4). Since activation of PI3K/Akt pathway in nonimmune cells, such as cardiomyocytes [34] or endothelial cells [35], could enhance cellular survival or inhibit oxidative stress, we further performed confocal imaging analysis to determine the location of p-Akt expression. The results showed that the majority of p-Akt expression was localized to CD206+ cells, a specific marker for M2 macrophages, in both control and BAY 60-6583 pretreated mice. In addition, the PI3K inhibitor attenuated the anti-inflammatory effects of BAY 60-6583 (Figures 5–7). These findings strongly suggest that BAY 60-6583 exerts its infarct-limiting effect not by acting on cardiomyocytes but possibly by acting on macrophages via a PI3K/Akt pathway.

It should be noted that a regulatory inflammation environment could promote macrophages phenotype switch to M2 subset [36]. Although our results strongly indicated BAY 60-6583 modulates macrophages phenotype switching after myocardial IR injury, there are several cell types other than macrophages which may mediate BAY 60-6583 induced cardioprotection, which warrants further studies. Koeppen and his colleagues [8] demonstrated that BAY 60-6583 protects heart from myocardial ischemia and reperfusion injury by acting on bone marrow derived cells. They found that BAY 60-6583 inhibited tumor necrosis factor *α* release of PMNs, which limited myocardial injury. van der Hoeven and his colleagues [37] showed that A_2B_R activation suppressed oxidase activity in neutrophils. Moreover, A_2B_R was also found in dendritic cells and lymphocytes. Studies are needed to define the roles of these cells during myocardial IR injury and the cardioprotection effects of A_2B_R.

In present study, we did not analyze the cardiac function after myocardial reperfusion injury. However, it is well known that histological determination of myocardial infarction has been widely used to estimate the cardiac injury in animal models [38] and utilized as the golden standard to validate novel imaging methods of infarction quantification, including cardiac MRI [39] and contrast echocardiography [40]. Moreover, using a cardiac MRI imaging technique, we have defined in our previous publication that left ventricular function is depressed proportionally to the size of infarction, which was determined by the same TTC staining technique in the present study [41]. Hence, it is reasonable to speculate that BAY 60-6583 could improve the cardiac function after reperfusion injury. However, further study is warranted to determine the role of A_2B_R activation during long-term ventricular remodeling after myocardial infarction.

In summary, our work demonstrates that the A_2B_R agonists reduce myocardial IR injury by inhibiting inflammatory responses in reperfused heart, probably by promoting macrophage phenotype switching to an anti-inflammatory M2 subset.

## Figures and Tables

**Table 1:** Perioperative heart rates.

Groups	Baseline	During ischemia	Reperfusion
Control	437 ± 20	479 ± 18^*∗*^	480 ± 20^*∗*^
IPC	438 ± 9	492 ± 8^*∗*^	494 ± 6^*∗*^
ATL + IPC	440 ± 8	486 ± 6^*∗*^	496 ± 5^*∗*^
BAY	431 ± 13	485 ± 11^*∗*^	481 ± 13^*∗*^
ATL + BAY	411 ± 10	503 ± 15^*∗*^	501 ± 11^*∗*^
Wort + BAY	433 ± 6	502 ± 9^*∗*^	501 ± 8^*∗*^

IPC: ischemic preconditioning; ATL: ATL-801; BAY: BAY 60-6583; Wort: wortmannin.

^*∗*^ *P* < 0.05 versus baseline.
